# Neurophysiological mental fatigue assessment for developing user-centered Artificial Intelligence as a solution for autonomous driving

**DOI:** 10.3389/fnbot.2023.1240933

**Published:** 2023-11-30

**Authors:** Andrea Giorgi, Vincenzo Ronca, Alessia Vozzi, Pietro Aricò, Gianluca Borghini, Rossella Capotorto, Luca Tamborra, Ilaria Simonetti, Simone Sportiello, Marco Petrelli, Carlo Polidori, Rodrigo Varga, Marteyn van Gasteren, Arnab Barua, Mobyen Uddin Ahmed, Fabio Babiloni, Gianluca Di Flumeri

**Affiliations:** ^1^Department of Anatomical, Histological, Forensic and Orthopaedic Sciences, Sapienza University of Rome, Rome, Italy; ^2^BrainSigns SRL, Rome, Italy; ^3^Department of Computer, Control, and Management Engineering, Sapienza University of Rome, Rome, Italy; ^4^Laboratory of Industrial Neuroscience, Department of Molecular Medicine, Sapienza University of Rome, Rome, Italy; ^5^Department of Civil Engineering, Computer Science and Aeronautical Technologies, Roma Tre University, Rome, Italy; ^6^Department of Enterprise Engineering, University of Rome Tor Vergata, Rome, Italy; ^7^Italian Association of Road Safety Professionals (AIPSS), Rome, Italy; ^8^Instituto Tecnologico de Castilla y Leon, Burgos, Spain; ^9^Academy for Innovation, Design and Technology, Mälardalens University, Västerås, Sweden; ^10^College of Computer Science and Technology, Hangzhou Dianzi University, Hangzhou, China

**Keywords:** road safety, simulated driving, mental fatigue, multimodal assessment, EEG index

## Abstract

The human factor plays a key role in the automotive field since most accidents are due to drivers' unsafe and risky behaviors. The industry is now pursuing two main solutions to deal with this concern: in the short term, there is the development of systems monitoring drivers' psychophysical states, such as inattention and fatigue, and in the medium-long term, there is the development of fully autonomous driving. This second solution is promoted by recent technological progress in terms of Artificial Intelligence and sensing systems aimed at making vehicles more and more accurately aware of their “surroundings.” However, even with an autonomous vehicle, the driver should be able to take control of the vehicle when needed, especially during the current transition from the lower (SAE < 3) to the highest level (SAE = 5) of autonomous driving. In this scenario, the vehicle has to be aware not only of its “surroundings” but also of the driver's psychophysical state, i.e., a user-centered Artificial Intelligence. The neurophysiological approach is one the most effective in detecting improper mental states. This is particularly true if considering that the more automatic the driving will be, the less available the vehicular data related to the driver's driving style. The present study aimed at employing a holistic approach, considering simultaneously several neurophysiological parameters, in particular, electroencephalographic, electrooculographic, photopletismographic, and electrodermal activity data to assess the driver's mental fatigue in real time and to detect the onset of fatigue increasing. This would ideally work as an information/trigger channel for the vehicle AI. In all, 26 professional drivers were engaged in a 45-min-lasting realistic driving task in simulated conditions, during which the previously listed biosignals were recorded. Behavioral (reaction times) and subjective measures were also collected to validate the experimental design and to support the neurophysiological results discussion. Results showed that the most sensitive and timely parameters were those related to brain activity. To a lesser extent, those related to ocular parameters were also sensitive to the onset of mental fatigue, but with a delayed effect. The other investigated parameters did not significantly change during the experimental session.

## 1 Introduction

Road transportation is an essential service of modern society relating to working activity and spare time. In particular, with respect to working activity, before the COVID outbreak, 70% of European citizens needed to commute to work, with more than 50% of them having <30 min one-way commuting time every day and another 25 of them having at least 30 min one-way commuting time every day (Passenger Mobility Statistics., 2023). Since the majority of people in Europe travel by private automobile (50% do so daily compared to only 16% who use public transportation and 12% who use bicycles) (Giménez-Nadal et al., 2022), time spent driving can be estimated to be a relevant part of daily activity. The trend is similar in the US where the Department of Transportation (Transportation Statistics Annual Report and Bureau of Transportation Statistics, 2023) estimates that 87% of persons use private vehicles for daily trips (both for work and spare time) and 91% commute to work using personal vehicles, with a total of 55 min spent driving per day.

Autonomous Driving (AD) could free humans from the burden of this daily driving routine. The level of driving autonomy is ranked using the acronym SAE (derived from the Society of Automotive Engineering) from level 0 – total manual driving – to SAE level 5 – fully autonomous. AD vehicles (SAE > 3) are supposed to be the main modality of driving in the future. In a survey from 2015 (Kyriakidis et al., 2015), AD was supposed to be commercially available in 2025 and participants seemed to be enthusiastic to have the possibility of performing other tasks instead of driving such as emailing, reading, and watching a movie. Today in 2023, partially and fully autonomous vehicles are on the market even if legislation must be refined with AD being a reality in just a few countries and with strict limitations. Apart from the cost, the major factor limiting the drastic adoption of AD is the one related to safety. In fact, AD vehicles need to be extremely accurate and reliable before totally entrusting them with the task of transporting people. This is especially true for highly automated vehicles (from SAE Level 3 to SAE level 5) which do not require user intervention in most (SAE 3 and 4) or every condition (SAE 5). But with lower levels of SAE (SAE 0 to 2), human intervention is required (Eriksson and Stanton, 2017; Vogelpohl et al., 2019), raising a concern in the opposite direction. This concern is related to the capacity of the driver to take control of the vehicle (i.e., takeover) when the Artificial Intelligence (AI) driving the car prompts the request. This capacity to timely intervene appears to be crucial with intermediate levels of autonomy, i.e., SAEs 1–3. In order to react properly to the takeover request, the drivers must be alert and focused on the surrounding environment (Huang et al., 2020; Weaver and DeLucia, 2022). This means that even if the drivers are not actually driving, they should be conscious that their intervention may be needed and for this reason, they should keep their focus on the driving. Many experimental pieces of evidence have reported that when performing a task, the automatization of different features may lead to mind-wandering and to a phenomenon called “out-of-the-loop” (Di Flumeri et al., 2019; Merat et al., 2019; Schnebelen et al., 2020). This was first noticed in the aviation field where autonomous piloting has long existed (Endsley and Kiris, 1995). Endsley and Kiris noticed that when supervising an automated process, pilots may enter a passive state, losing information coming from the environment and lacking situational awareness. In the automotive field, with increasing automation, the driver acts as a supervisor of the automation system, potentially increasing the chance of being “out-of-the-loop” and therefore increasing the risk of accidents for themselves and others. In other words, monitoring the state of the drivers in an effective and reliable way will be as crucial as monitoring the surroundings through traditional cameras, radar, and LiDAR systems.

In fact, in order to reduce the risk of humans failing the takeover request, the AI driving the cars should be aware of the state of the drivers (i.e., “in-” or “out-of-the-loop”) and able to modulate their activity to keep a constant and functional level of engagement (Aricò et al., 2016; Di Flumeri et al., 2019), or at least to provide feedbacks and suggestions accordingly (Boelhouwer et al., 2020). Also, the drivers could be “in-the-loop” without actually being in the proper mental state for a driving task when a takeover intervention is requested. For example, the drivers could be over-stressed or overloaded from previous activities other than driving or they could just be drowsy or fatigued. Even if drivers are just supervising the AD system, their mental resources to face the takeover request could be insufficient and thus the drivers might not be completely aware of the situation and so not ready to intervene/react promptly. In other words, the Artificial Intelligence playing autonomous functions in the vehicle has to be user-centered.

In this scenario, monitoring drivers' mental state appears to be crucial to prevent fatal and/or non-fatal accidents and this is true for AD as well as human driving. Indeed, the automotive market is already developing driver monitoring systems to detect improper mental states while driving (Doudou et al., 2020). These systems are mainly focused on the detection of fatigue, including its degradation to drowsiness and inattention, which are two of the most impacting impairing factors while driving. These systems belong to three categories of driver-monitoring devices: (i) driving behavior-based, (ii) driver behavior-based, and (iii) driver physiological signal-based. Since the study focuses on the assessment of mental fatigue, a short description of the previous categories with respect to fatigue assessment is provided below:

i. Driving behavior-based systems have been developed by several vehicle manufacturers with the aim of using information coming from the vehicle to detect any driving pattern related to a drowsy or fatigued state. The measures collected for this kind of detection focus mainly on different driving parameters that have been experimentally demonstrated to be related to the fatigue and drowsy levels of drivers such as steering wheel angle (Fairclough and Graham, 1999; Eskandarian and Mortazavi, 2007; Borghini et al., 2014), vehicle deviation and position (Ingre et al., 2006; Forsman et al., 2013), and vehicle speed and acceleration (Fairclough and Graham, 1999; Arnedt et al., 2001; Chen et al., 2015).
ii. Drivers' behavior-based systems aim to directly monitor drivers' activity (driver behavior-based) in order to detect any symptoms of a fatigued or drowsy state. Drivers' behaviors are usually monitored using a camera and thus this approach is referred to as a video-based measure. Research in this area mainly focused on eye movements [eyeblink rate – EBR (Papadelis et al., 2007), eyeblink duration – EBD (Häkkänen et al., 1999; Danisman et al., 2010; Shekari Soleimanloo et al., 2019), percentage of eye closure – PERCLOS (Sommer and Golz, 2010), facial expression (Fan et al., 2007; Knapik and Cyganek, 2019) and head position (Brandt et al., 2004; Ghourabi et al., 2020)].
iii. It is known that internal mental states are reflected in changes in several physiological parameters that can be measured to monitor drivers' conditions. In contrast to the other two already described families, such systems include large, hefty amplifiers and preprocessing components in the acquisition module. Since there are typically many cables between the electrodes and the acquisition component, connecting wires might be challenging. These factors lead to a lengthy preparation period while monitoring signals. Additionally, the user's ability to move is constrained by cable restrictions. Fatigue detection based on these technologies is therefore usually aimed at providing a reference measure and result to be only applicable in a laboratory environment even if technological advances are starting to allow the recording of good quality signals using wearable devices out of controlled environments nowadays. There are several physiological measures to monitor drivers' mental states in a laboratory condition and the most used are Electroencephalography (EEG), Electrooculography (EOG), Electrocardiography (ECG), Photopletismography (PPG), and Electrodermal Activity (EDA). EEG measures brain cortical activity by placing electrodes on the scalp. Its high temporal resolutions (milliseconds) would make it the best candidate to intervene timely to prevent accidents due to an improper mental state to drive (Islam et al., 2020). Indeed, several studies highlighted the possibility of monitoring vigilance and drowsiness levels with EEG (Zhang et al., 2016; Guo et al., 2017; Majumder et al., 2019; Stancin et al., 2021). There is broad agreement on the fact that low-frequency rhythms are indicative of a level of a fatigued or drowsy state even if some studies focused on Alpha rhythm (Fujiwara et al., 2018; Di Flumeri et al., 2022) while others investigated Theta and Delta (Nguyen et al., 2017; Arefnezhad et al., 2022). EOG monitors eye behavior using electrodes placed near the ocular bulb to detect movements and blinks (Barea et al., 2002). Given the constraint of such a method, EOG can be estimated from EEG signal (Sciaraffa et al., 2021; Simonetti et al., 2023). This approach reduces the invasiveness of EOG monitoring while keeping the information suitable for estimating the physiological parameters relevant to fatigue and drowsiness detection. The measures obtained with this approach are the same as the video-based measures. Another source of information regarding drivers' states is represented by the analysis of parameters estimated from the autonomic response. In particular, ECG and PPG signals, which are related to heart activity, and EDA which is related to skin sweating, are relatively easy to record and they can bring relevant information about the fatigue and drowsy state. Heart Rate (HR) is one of the most common features extracted from ECG and it represents the number of heart beats in a temporal unit. The variation over time of the distance between two heart beats, namely, Heart Rate Variability (HRV), has been demonstrated to be correlated with the state of drowsiness and it was found to decrease in sleepiness compared to alertness (Fujiwara et al., 2018; Alaimo et al., 2020). From the EDA signal, it is possible to extract two features, the Skin Conductance Level (SCL) and the Skin Conductance Response (SCR) that were found to be correlated with drivers' mental fatigue and drowsiness levels. The research on these parameters is more immature with respect to the previous ones; however, there are some findings about a possible relation between skin conductance variations and mental fatigue (Geldreich, 1939; Bundele and Banerjee, 2009).

Thus, there is the feeling that all the listed neurophysiological parameters are, to different degrees, linked to mental fatigue. Their multimodal analysis could increase the sensitivity and reliability of a monitoring system in detecting the onset of fatigue. They would become a valuable resource for developing in-car human-machine interfaces that allow the vehicle to recognize the user's state and act accordingly. It could be argued that, as introduced, these neuro-technologies are still far from being deployable seamlessly in a real scenario. However, their application would be paramount, especially considering the limitations of the two other strategies: on one hand, the driver behavior-based systems have been demonstrated to be sometimes not reliable mostly because of lighting conditions (Sahayadhas et al., 2012) and a further decrease in reliability was found in the real environment compared to control environment (Philip et al., 2005); on the other hand the driving behavior-based systems, apart from arguable reliability as well as depending on road geometric characteristics and traffic conditions (Sahayadhas et al., 2013), are not suitable for vehicles with increasing autonomy (SAE > 2) since in this case the driving behavior will depend on AI and so is not linked to the drivers' state. To achieve optimal decision-making on AI driving the car, it should be fed with data collected from the drivers. Dedicated algorithms would perform analysis of the information coming from sensors in order to detect the drivers' suitability for the driving activity. In order to do this, a machine-learning approach is usually adopted. Several studies have demonstrated the feasibility of using neurophysiological parameters to recognize drowsiness occurrence using a machine learning approach (Leng et al., 2015; Choi et al., 2017; Kundinger et al., 2020). In these studies, several neurophysiological parameters such as EEG, EDA, heart-related measures, and temperature are used to recognize fatigue and drowsiness occurrence in the drivers. In this approach, supervised learning is used to train the machine learning model. Ground truth is usually represented by subjective ratings of the drivers or by image processing analyzing facial expressions and eye closure. Classification models like these have been demonstrated to be reliable in recognizing drowsy and fatigued states.

In any case, none of the cited studies handle this topic in a holistic way, i.e., by simultaneously considering all the listed biosignals in order to provide a clear overview of which of them are the most informative and timely. Also, in the majority of the studies, participants had to drive for hours, sometimes under conditions of sleep deprivation, and the two extreme “boundaries” of the experience, i.e., the period when the driver was fully awake vs. the period when the driver was highly drowsy, were compared in terms of neurophysiological markers. This comparison undoubtedly allows us to obtain significant outcomes since the two conditions are very different, but at the same time, it is still far from daily situations and requirements since mental impairment and unsafe driving behaviors begin long before fatigue becomes extreme.

In this study, the main aim is to investigate whether it is possible, and by which parameters, to detect the onset of fatigue at a very early stage since anticipation will be crucial for developing safer vehicles. Most of the previous studies found in the literature focused either on vigilance or severe sleepiness thus not filling the gap existing between the two. Indeed, fatigue could be interpreted and defined as a precursor of the drowsy state. On the other hand, a decrease in vigilance, often a topic of investigation in this field, could be one of the early symptoms of a fatigued mental state, which is recognized to reduce the ability to perform a task adequately (Shen et al., 2006; Shahid et al., 2010). Not surprisingly, vigilance decrease and fatigue increase are often linked to similar physiological reactions, such as the increase in alpha activity and eye blink rate (Tejero Gimeno et al., 2006; Borghini et al., 2014). In other words, all these mental states can be considered a continuum (alertness, fatigue, and drowsiness).

To achieve the intended purpose, 26 professional drivers were engaged in a simulated monotonous driving task. A multimodal approach was adopted in order to understand which physiological parameters could better detect the onset of fatigue occurrences while driving. Data collection of the physiological variables was performed using high-quality wearable devices in order to collect reliable data without interfering with the driving activity. EEG, EOG, EDA, and PPG signals were then acquired from healthy volunteers and derived features were used to estimate their level of mental fatigue. In particular, in terms of EEG data, a previously validated neurometric index of mental fatigue/drowsiness derived index (Di Flumeri et al., 2022; Ronca et al., 2022) was used. Performance in a realistic secondary task and subjective measures were also collected to validate the experimental design and to support the eventual findings.

## 2 Materials and methods

### 2.1 Participants and experimental setup

Twenty-six (26) professional drivers (26 men, 37.7 years old ± 10.8), with normal or corrected-to-normal vision were recruited to take part in the study. The experiment was conducted following the principles outlined in the Declaration of Helsinki of 1975, as revised in 2008, and it was approved by the Sapienza University of Rome and Roma Tre Ethical Committee. Experiments took place in the afternoon to ease a higher level of mental fatigue.

### 2.2 Experimental protocol

The research tool was a driving simulator constituted of a real car seat, a real dashboard with steering wheel, manual gearshift and pedals, and a 3-monitor-based display with a 160° view.

After an initial training phase, the neurophysiological activity in a resting state was collected. Participants were asked to sit and close their eyes for 1 min (EC condition). Then they were instructed to look at the main monitor for 1 min without performing any task (EO condition, EO1). Subsequently, participants were provided for the first time with questionnaires (described below).

**Figure 1:**
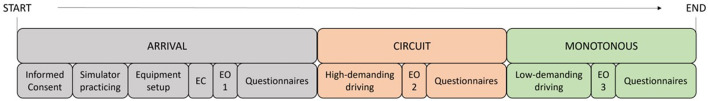
Experimental design to induce fatigue. The 45 mins Monotonous task was preceded by a high demanding Circuit driving task which lasted 15 mins. Asterisks indicate significance: *p < 0.05; **p < 0.01; ***p < 0.001.

Then, participants had to drive in two simulated environments, a challenging and a monotonous one, in a fixed order, according to what was suggested by scientific literature (Thiffault and Bergeron, 2003; García et al., 2010). Specifically, fatigue is supposed to be promoted by an intense task load, followed by a low stimulating and monotonous task (Thiffault and Bergeron, 2003; García et al., 2010). The protocol we designed was constituted of the first high-demanding driving simulation which aimed to challenge the mental resources of the participants. On the contrary, the second simulated environment was designed to be extremely easy and repetitive. The first simulated environment consisted of a 15-min high-demanding circuit driving task. This task was designed to challenge participants' driving abilities in order to increase the probability of fatigue episodes in the following easy and monotonous driving task. After the Circuit task, the Eyes Open condition (EO2) was performed again and participants were provided with the second round of questionnaires. The monotonous driving task lasted 45 min and it consisted of driving in an easy and repetitive path, reproducing urban road infrastructure, without traffic. The speed limit was set at 40 km/h. Participants were asked to perform a secondary task that aimed to collect their Reaction Times (RT) (Blanco et al., 2006; Collet et al., 2009) while driving in this monotonous environment. During this secondary task, a fake engine failure alarm was presented both acoustically and visually. Participants had to address the issue by pushing a button on the steering wheel to turn off the alarm. The reaction time needed to push the button was taken as the performance of the secondary task. At the end of the monotonous driving task, the last Eyes Open condition (EO3) and questionnaire phase were performed. A scheme depicting the entire experimental protocol is given below (Figure 1).

### 2.3 Subjective assessment

Two questionnaires were adopted to validate the experimental designs collecting perceived feelings of fatigue and drowsiness. Karolinska Sleepiness Scale (KKS) (Kaida et al., 2006) and Chalder Fatigue Scale (Chalder) (Cella and Chalder, 2010) were presented at the arrival and after each driving task for fatigue rating. From a conceptual and psychological point of view, mental fatigue and drowsiness are slightly different even if they are usually considered just as two different degrees of intensity on a scale from alertness to sleepiness (Kamran et al., 2019). Because of that, the redundant choice to ask the participants to fill out both questionnaires was made because, being contiguous phenomena, they are often hard to distinguish between each other, especially if considering the poor sensitivity of subjective measures.

#### 2.3.1 Karolinska Sleepiness Scale

KSS (Kaida et al., 2006) asks participants to rate their current state of sleepiness on a scale from 1 (extremely alert) to 9 (extremely sleepy – fighting sleep). This scale measures the subjective level of sleepiness at a particular time during the day and therefore it is sensitive to fluctuations.

#### 2.3.2 Chalder Fatigue Scale

Chalder questionnaire (Cella and Chalder, 2010) asks participants to answer several questions about fatigue-related symptoms on a scale from 0 (none) to 3 (very high). In the original form, Chalder questions refer to two different dimensions called “physical symptoms” and “mental symptoms.” Given the focus of this study (i.e., mental fatigue), only the questions related to this dimension were used (questions from 9 to 14 of the original questionnaire).

### 2.4 Behavioral assessment

During the Monotonous condition, nine fake engine failure alarms were presented at fixed intervals of around 5 min (all the intervals were different to avoid any anticipation/habituation effect). The participants' task was to push a button on the steering wheel to solve the issue. The assumption was that if the participants were impaired by any not-proper-to-drive mental state, their performance in this task would decrease (slower, i.e., higher reaction times) (Blanco et al., 2006; Collet et al., 2009).

### 2.5 Neurophysiological assessment

#### 2.5.1 Electroencephalographic signal

EEG signal was collected using the Mindtooth device (developed and validated during Mindtooth Project, GA 950998) (Sciaraffa et al., 2022a,b). It consists of eight Ag/AgCl water-based electrodes placed according to the International 10–20 system (AFz, AF3, AF4, AF7, AF8, Pz, P3, and P4) plus ground and reference electrodes placed on mastoids. The device has been validated and is capable of recording the EEG signal extremely accurately (Sciaraffa et al., 2022a). The sampling frequency was 125 (Hz). To remove interferences due to mainline power interference, a 50-Hz notch filter was applied. The EEG recordings were also band-pass filtered [low-pass filter cut-off frequency: 40 (Hz), high-pass filter cut-off frequency: 2 (Hz)]. Subsequently, the Reblinca (Di Flumeri et al., 2016) algorithm was used to remove eyeblink artifacts, while for other sources of artifacts, dedicated algorithms of the EEGLAB toolbox (Brunner et al., 2013) were applied. We estimated an average of 21% (± 22.51) of data loss due to artifact rejection in both EEG and EOG (EEG-derived) signals. In detail, the ICA-processed signal has been separated into 1-s-long epochs and three criteria have been applied in order to recognize artifactual data. Firstly, EEG epochs with a signal amplitude exceeding ±80 mV (Threshold criterion) were labeled as “artifacts”. Then, each EEG epoch was interpolated in order to check the slope of the trend within the considered epoch (Trend estimation criterion). If such a slope was higher than 20 mV/s, the considered epoch was marked as “artifact.” Finally, the signal sample-to-sample difference (Sample-to-sample criterion) was analyzed: if such a difference, in terms of absolute amplitude, was higher than 25 mV, i.e., an abrupt variation (no-physiological) happened, the EEG epoch was marked as “artifact”. In the end, the EEG epochs marked as “artifacts” were removed from the EEG dataset with the aim to have a clean EEG signal to perform the analyses.

The Global Field Power (GFP) was calculated from the artifact-free EEG with a focus on the EEG frequency band for the mental state of interest, which was the Alpha band (Di Flumeri et al., 2022). The GFP was chosen because it describes brain EEG activity with the advantage of representing, in the time domain, the degree of synchronization of a specific cortical region of interest in a specific frequency band (Skrandies, 1990). According to the Individual Alpha Frequency (IAF) value (Klimesch, 1999), the Alpha band was computed for each participant. Since the Alpha peak is mainly prominent during rest conditions, the subjects were asked to keep their eyes closed for a minute before starting the experiment. Such a condition was then used to estimate the IAF value specifically for each participant. Consequently, an EEG “strict” Alpha band was defined as Alpha = (IAF - 1) : (IAF + 1) Hz. This definition of the Alpha band is more restrictive (thus “strict”) compared to the vast majority of Alpha band definitions that can be found in scientific literature, which is (IAF-2) : (IAF + 2) Hz. This approach was selected according to Klimesch (2012), who demonstrated that a tighter band around the IAF can be considered Alpha to avoid the impact from closer EEG frequency band (Theta and Beta) variations on the observed phenomena in the Alpha band. The GFP was calculated over all the EEG parietal channels for each epoch using a Hanning window of the same length of the considered epoch (1 s length, which means 1 Hz of frequency resolution). The EEG was used to compute a Mental Drowsiness index (MDrow) (Di Flumeri et al., 2022), which is based on the increased Alpha GFP in parietal regions.

#### 2.5.2 Electrooculographic signal

The electrooculographic (EOG) signal was derived from EEG data. The vertical EOG pattern was estimated by analyzing the EEG AFz channel. This analysis was based on the application of a customized version of the Reblinca method (Di Flumeri et al., 2016) to isolate and identify the eyeblinks. The Eyeblinks Rate (EBR), Eyeblinks Amplitude (EBA), and Eyeblinks Duration (EBD) parameters were then estimated for each minute during the monotonous driving task.

#### 2.5.3 Electrodermal activity and heart rate

The Empatica E4 wristband (Empatica, Massachusetts, United States) was adopted for both EDA and PPG signals with a sampling frequency of 4 and 64 Hz, respectively. The device was placed on the participants' non-dominant arms. The EDA signal was first low-pass filtered with a cut-off frequency of 1 Hz and subsequently processed with the Ledalab suite (Bach, 2014), a dedicated open-source toolbox implemented within the MATLAB (MathWorks, Natick, Massachussets) environment for EDA processing. EDA-derived tonic (Skin Conductance Level, SCL) (Braithwaite et al., 2013) was estimated with the continuous decomposition analysis (Benedek and Kaernbach, 2010). The SCL represents the slow-changing component of the EDA signal and it is recognized to bring information regarding the global arousal of an individual. A 60-second time resolution window was used to estimate the EDA components and the other neurophysiological parameters adopted in this study. Given the low sampling frequency of Empatica E4 wristband EDA sensors (4 Hz), Skin Conductance Response, the fast-changing component of EDA usually linked to reactions to a single stimulus, was not taken into consideration for the low reliability obtainable with the adopted device (Ronca et al., 2023).

Empatica E4 device was also employed for PPG recording to derive the Heart Rate (HR) and Heart Rate Variability (HRV) parameters. PPG signal was filtered using a 5th-order Butterworth band-pass filter (0.4 Hz) in order to reject the continuous component and the high-frequency interferences, such as that related to the mains power source. An additional reason to apply this filtering was to emphasize the “pulse” process of the PPG signal (Goovaerts et al., 1976; Pankaj et al., 2022). At this point, the “pulses,” i.e., the phenomena related to the heartbeats, were detected by the Pan-Tompkins algorithm (Pan and Tompkins, 1985). Finally, the temporal distance (inter-beats interval, IBI) between consecutive beats was measured to estimate the HR values every 60 s. The IBI signal was also analyzed to estimate the Heart Rate Variability (HRV). In particular, the HRV was analyzed in the frequency domain by computing the Lomb-Scargle periodogram (Ruf, 1999) of the IBI signal. Analysis has shown that the Lomb-Scargle periodogram can produce a more accurate estimate of the Power Spectrum Density (PSD) than Fast Fourier Transform methods for typical HR data (Simonetti et al., 2023). Since the HR data are unevenly sampled data, another advantage of the Lomb-Scargle method is that in contrast to Fast Fourier Transform-based methods, it is able to be used without the need to resample and de-trend the RR data (Clifford and Tarassenko, 2005). According to scientific literature, the PSD of the HRV signal was computed over the Low (LF: 0.04 ÷ 0.15 Hz) and the High Frequencies (HF: 0.15 ÷ 0.4 Hz), and then the LF/HF ratio was computed as a relevant indicator of HRV (Ori et al., 1992). The HRV analysis was performed by means of the HRVAS MATLAB suite (Ramshur, 2010). When processing EDA and PPG signals, artifacts due to movements were corrected interpolating between two portions of good-quality data, thus no data loss was found for these datasets.

### 2.6 Statistical analysis

In order to compare the data collected from different participants at a group level, data were normalized by means of Z-score normalization, with the exception of the MDrow index, which is directly comparable between participants, and SCL which was normalized using the minimum and the maximum values recorded during the experimental session.

Gaussian distribution of the continuous variables (Reaction Times -RT- of the secondary task, EEG, EOG, EDA, and PPG-derived parameters) was verified using the Shapiro-Wilk Test. If normality was confirmed, a parametric test was performed; otherwise, a non-parametric test was used. Questionnaires were analyzed with the non-parametric test. In particular, when comparing three conditions (within the same subject), repeated measures of ANOVA and Friedman test were performed as parametric and non-parametric tests, respectively, for the overall effect. In the case of a significant overall effect, pairwise comparisons between conditions were performed by means of a *post-hoc* test. The reported “p” parameter of significance was always corrected for multiple comparisons by the Bonferroni-Holm method.

We refer to the experimental conditions as “Arrival” (data collected at participants' arrival), “Circuit” (data about the first driving task in the circuit), and “Monotonous” (data about the second driving task in the monotonous environment). EO conditions will be referred to as “EO1” (EO collected at participants' arrival), “EO2” (OE collected just after the Circuit driving task), and “EO3” (OE collected just after the Monotonous driving task). RT, EEG, EOG, EDA, and PPG-derived parameters measured during the monotonous driving task were divided into three segments of 15 min each and averaged (1°, 2°, and 3° segments).

After this first statistical analysis of the entire group, two different behaviors emerged among participants. Looking at the performance in the secondary task (i.e., reaction times), it appeared that some participants improved their performance along the task while others did not. We hypothesized that during a 45-min easy secondary task, performance should improve with time (Blanco et al., 2006; Collet et al., 2009). If this did not happen, it could be attributed to the occurrence of fatigue phenomena. Therefore, the sample group was split into two subgroups according to their behavioral performance. In particular, the difference between z-scored RT during the third and the first segment was computed for each participant. Those who showed a positive value (i.e., slower reaction times in the third segment of the monotonous driving task) were assigned to Group 1 (fatigued, *n* = 12), while those who showed a negative value (i.e., faster reaction times in the third segment) were assigned to Group 2 (not fatigued, *n* = 14). Section 3 first describes the whole sample group (Within-subjects Analysis) and then the analysis of the two groups separately (Between-subjects Analysis).

## 3 Results

### 3.1 Validation of experimental design — Subjective reports

#### 3.1.1 Karolinska Sleepiness Scale

**Figure 2:**
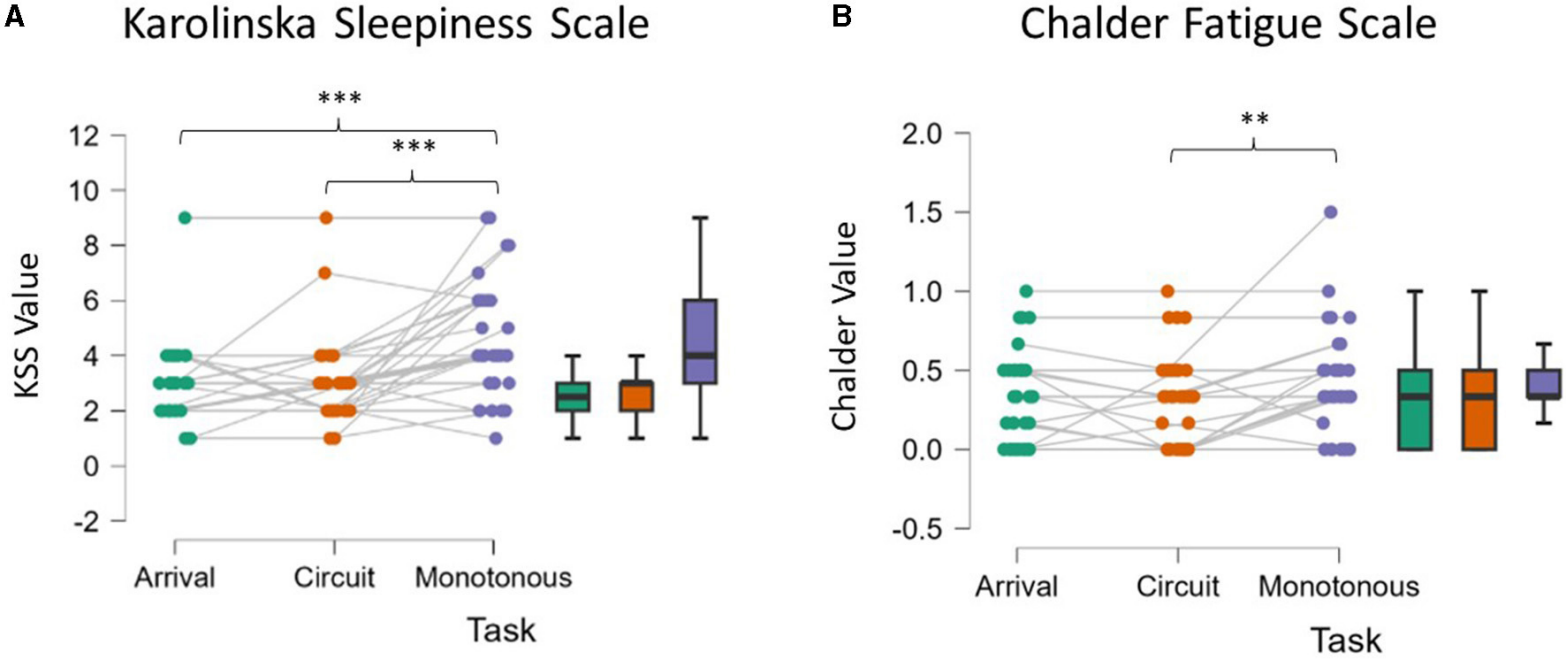
Self-report of Karolinska Sleepiness Scale **(A)** and Chalder Fatigue Scale **(B)**. After the Monotonous driving task participants reported higher level of sleepiness compared to their Arrival and after the Circuit **(A)**. Chalder Fatigue Scale after the Monotonous task resulted higher than Arrival **(B)**. Asterisks indicate significance: *p < 0.05; **p < 0.01; ***p < 0.001.

KSS questionnaire analysis showed an overall significant effect, with the level of perceived sleepiness increasing across the protocol (ANOVA, *p* < 0.001). As shown in Figure 2A, the *post-hoc* tests showed that after the Monotonous driving task, participants reported higher levels of sleepiness compared to both moments of the Arrival and after the Circuit driving task (*p* < 0.001).

#### 3.1.2 Chalder Fatigue Scale

Chalder Fatigue Scale analysis revealed significantly higher levels of perceived mental fatigue (ANOVA *p* = 0.006, Figure 2B). The *post-hoc* analysis pointed out that the fatigue experienced after the Monotonous driving task was significantly higher with respect to the Circuit driving task (*p* = 0.006), and almost significant with respect to the Arrival (*p* = 0.069), while no significant differences arose from the comparison between Circuit and Arrival (*p* = 0.305).

### 3.2 Within-subjects analysis

#### 3.2.1 Behavioral analysis

**Figure 3:**
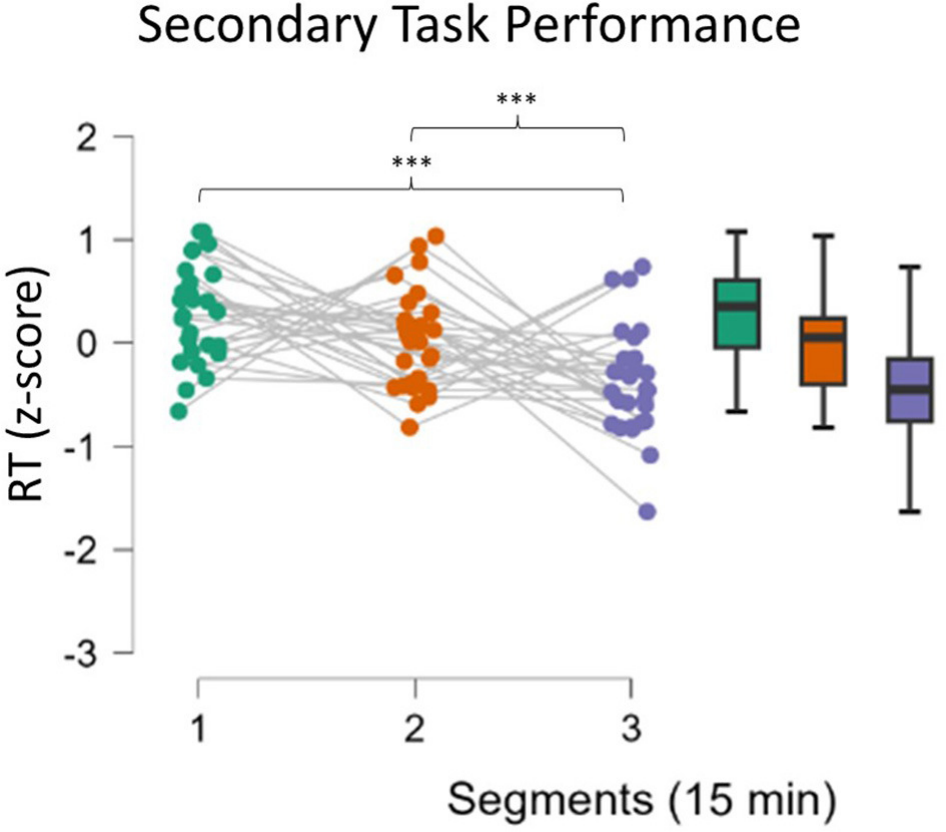
Analysis of reaction times in the secondary task. Participants showed increased performance (lower reaction times) in the third segment compared to the first two segments. Asterisks indicate significance: *p < 0.05; **p < 0.01; ***p < 0.001.

Performance of the secondary task improved with time during the Monotonous driving task (ANOVA, *p* < 0.001, Figure 3). Reaction times during the third segment decreased significantly compared to both the first and second segments (respectively, *p* < 0.001 and *p* = 0.02).

#### 3.2.2 Neurophysiological analysis

##### 3.2.2.1 EEG assessment

**Figure 4:**
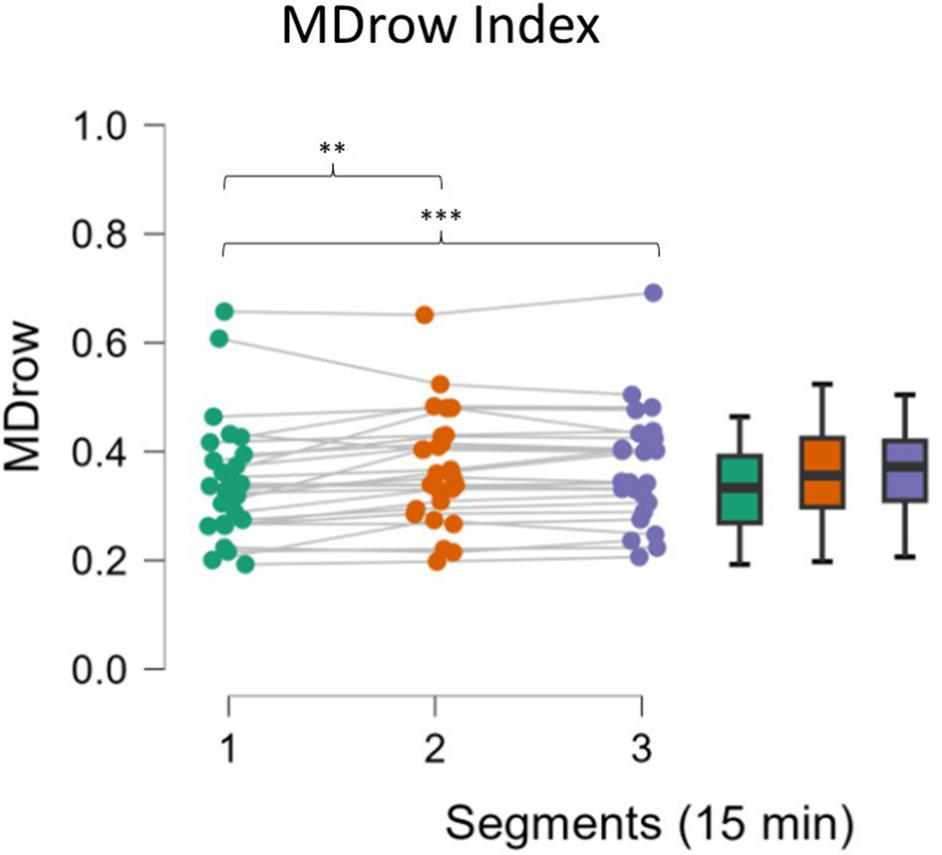
MDrow along the three segments. The level of MDrow increased significantly in the second and third segments compared to the first one. Asterisks indicate significance: *p < 0.05; **p < 0.01; ***p < 0.001.

MDrow EEG-based index was investigated during the three 15-min segments of the Monotonous task. An overall significant increase (*p* < 0.001) has been highlighted over time. As shown in Figure 4, compared to the first segment, statistical analysis revealed a higher MDrow in the second (*p* = 0.003) and third segments (*p* = 0.001), while no differences arose from the comparison between the second and third (*p* = 0.679).

##### 3.2.2.2 EOG assessment

**Figure 5:**
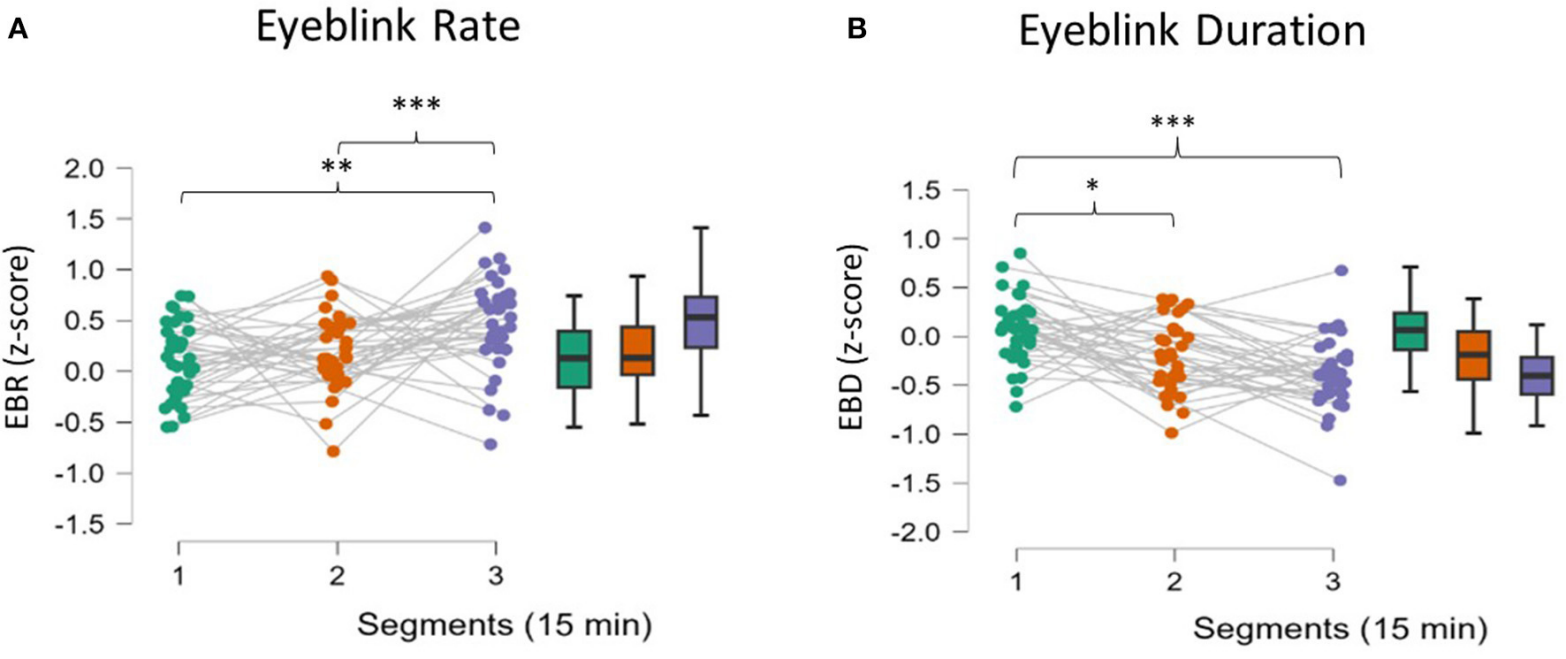
**(A, B)** Eyeblink behavior analysis. Participants showed increased EBR in the third segment compared to the first. EBD decreased significantly in each of the three segments. EBA did not change significantly during the experimental session. Asterisks indicate significance: *p < 0.05; **p < 0.01; ***p < 0.001.

EBR, EBD, and EBA were estimated from the EOG signal. Figure 5A shows the analysis of EBR, which proved to be significant (ANOVA, *p* = 0.004). *Post-hoc* comparisons revealed an increase in EBR in the third segment compared to the first (*p* = 0.003) and second segments (*p* = 0.001). Also, EBD proved to be significant after the analysis (ANOVA, *p* < 0.001, Figure 5B). During the second and third segments, participants showed decreased EBD compared to the first segment (respectively, *p* = 0.03 and *p* < 0.001). EBA analysis showed no significant effect (ANOVA, *p* > 0.05).

##### 3.2.2.3 EDA and HR assessment

EDA signal was used to estimate the SCL. Statistical analysis revealed no significant effect on SCL (ANOVA, *p* = > 0.05).

From the PPG signal, HR, HRV, HRV-LF, and HRV-HF were estimated, but statistical analysis highlighted no significant effect on these parameters (ANOVA, *p* > 0.05).

### 3.3 Between-subjects analysis

#### 3.3.1 Behavioral analysis

**Figure 6:**
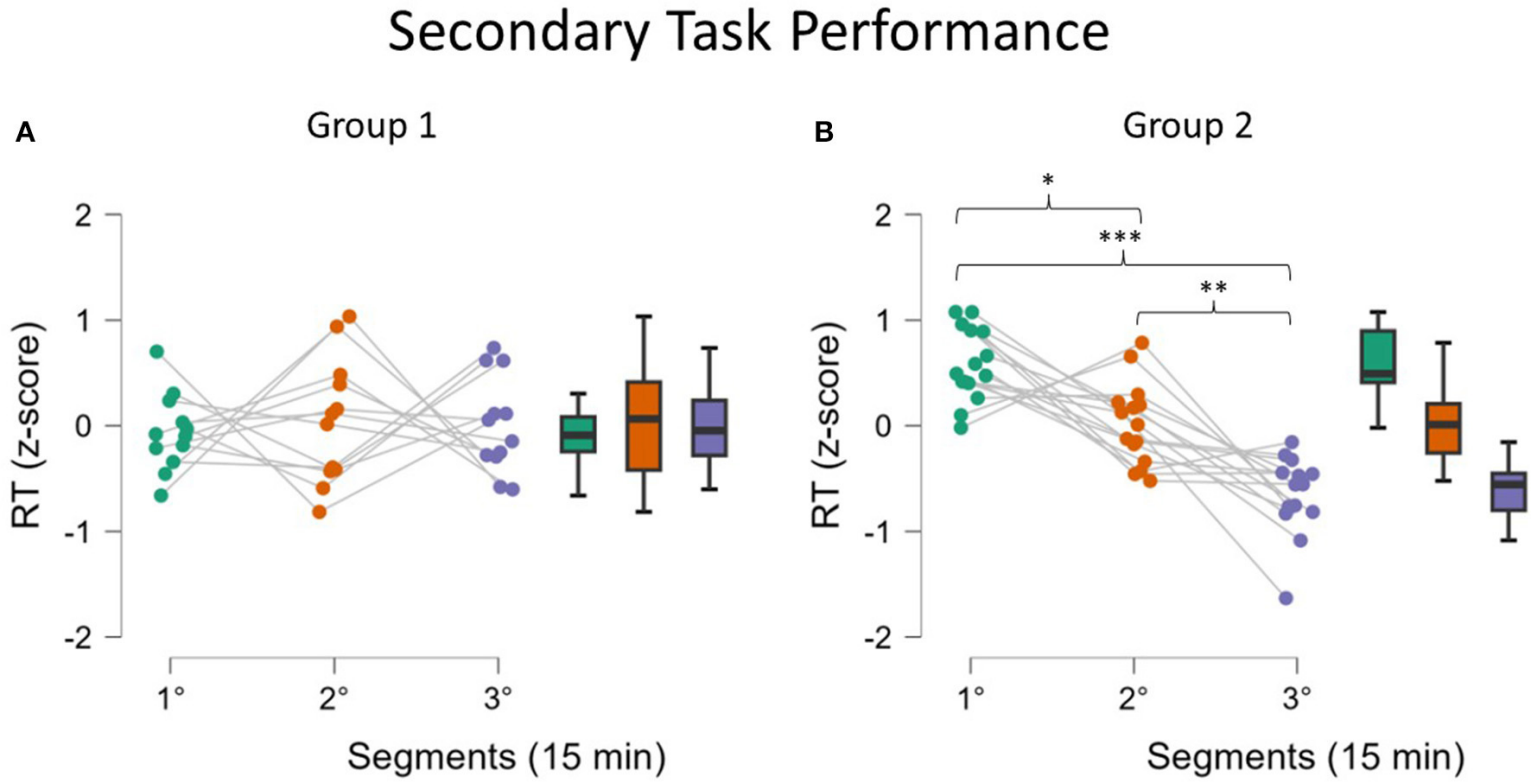
Reaction times in the secondary task for the two Groups. Analysis showed that Group 1 (**A**, fatigued) did not improve the performance during the task. On the contrary, Group 2 (**B**, not fatigued) improved the performance (lower reaction times). Asterisks indicate significance: *p < 0.05; **p < 0.01; ***p < 0.001.

Reaction times in the secondary task were used to split the participants into two groups, as described in the methodology. The ANOVA performed by including the “Group” variable confirmed the presence of such a moderating effect since a significant interaction between factors “Segments” and “Group” was found (*p* < 0.001). As shown in Figure 6, the performance of Group 1 (Figure 6A) did not improve along the Monotonous driving task, while for Group 2 (Figure 6B), statistical analysis showed an improvement in performance in the second and third segments compared to the first (*p* = 0.02 and *p* < 0.001, respectively). Further improvement in performance was found in the third segment compared to the second (*p* = 0.006).

#### 3.3.2 Neurophysiological analysis

##### 3.2.3.1 EEG assessment

**Figure 7:**
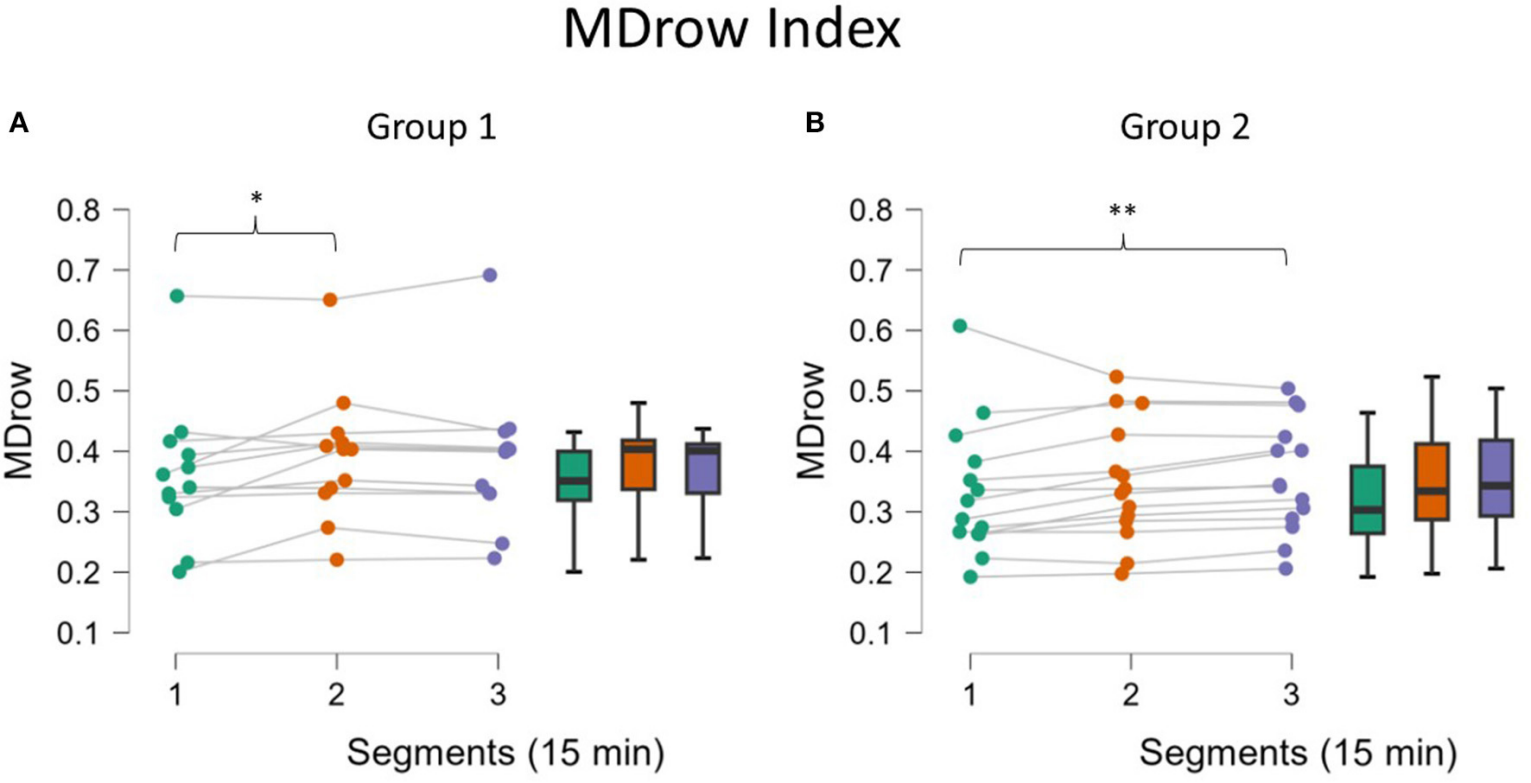
MDrow along the three segments for the two Groups. Analysis showed that for Group 1 (**A**, fatigued) the MDrow increased in the second segment while for Group 2 (**B**, not fatigued) the increase was found in the third segment. Asterisks indicate significance: *p < 0.05; **p < 0.01; ***p < 0.001.

Since the EEG-based MDrow index distributions are not Gaussian, the non-parametric tests were used; therefore, it was not possible to investigate the interactive factor but only the effect on the single Group. For Group 1, a significant increase of the MDrow level in the second segment compared to the first (*p* = 0.04, Figure 7A) was found. For Group 2, the increase was found between the first and third segments (*p* = 0.004, Figure 7B).

##### 3.2.3.2 EOG assessment

**Figure 8:**
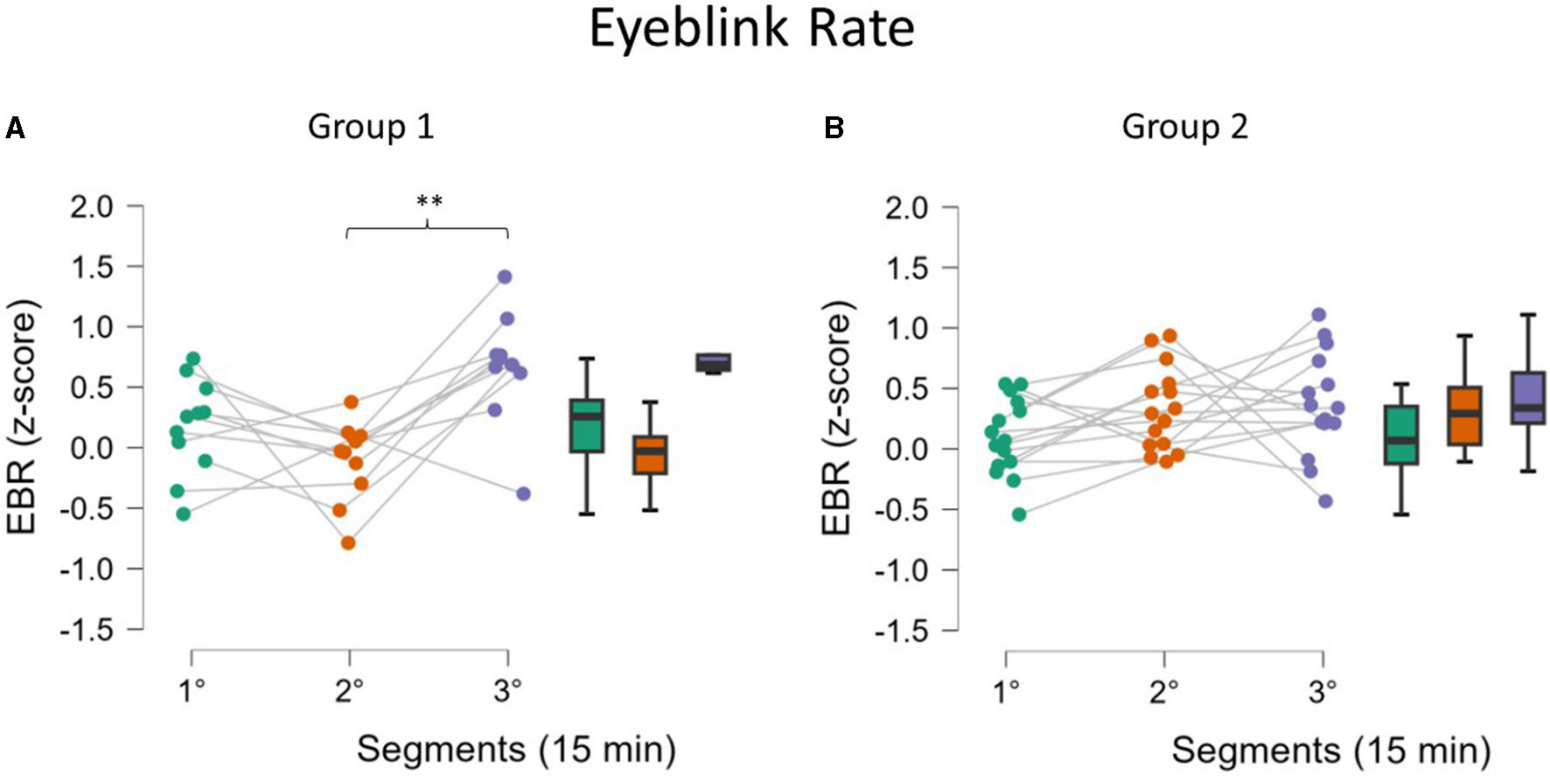
EBR along the three segments for the two Groups. Analysis revealed that for Group 1 (**A**, fatigued) there was a significant increase in the third segment, while this was not found for Group 2 (**B**, not fatigued). Asterisks indicate significance: *p < 0.05; **p < 0.01; ***p < 0.001.

EBR statistical analysis showed a different effect on the two Groups (ANOVA interaction effect between the factors Segments and Groups, *p* = 0.01, Figure 8). *Post-hoc* test confirmed an increase in EBR for Group 1 in the third segment compared to the second segment (*p* = 0.002, Figure 8A). For Group 2, no significant change was observed (Figure 8B). No significant effect was observed on EBD and EBA.

##### 3.2.3.3 EDA and HR assessment

None of the autonomic parameters (EDA, HR, HRV, HRV-LF, and HRV-HF) proved to be significant after the analysis of the interaction between Segments and Groups factors (ANOVA, *p* > 0.05).

In conclusion, here below it is reported a table (Table 1) summarizing all the results obtained over the full group, that is, the Within-subject Analysis, by reporting the overview of the neurophysiological features' behavior, whether each feature showed a significant effect, and in this case, if it decreased or increased with fatigue.

**Table 1:** In the table are summarized the results of the Within-subject Analysis of the monotonous driving task.

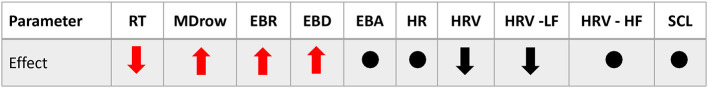

Upper and lower red arrows indicate respectively a significant (*p* < 0.05) increase or decrease of the mentioned parameter. A black arrow indicates a tendency (0.05 > *p* > 0.1) to an increase or decrease (upper or lower), while a black circle indicates that the parameter did not change during the task.

## 4 Discussions

The current automotive scenario is transitioning from manual to autonomous driving, which is promoted by technological advancement in terms of sensing technology and Artificial Intelligence. Manufacturers have been working on developing technologies that can “read” the traffic situation, “decode” the road infrastructure, and “sense” the surroundings, in order to make the AI vehicle able to make the proper decisions in a safe way. However, during this transition phase, the driving responsibility will be still shared between the human driver and the AI. Even at the higher levels of autonomy, i.e., when the AI is in charge of the majority of driving-related tasks, the driver has to supervise the AI's behavior and properly intervene when needed, i.e., takeover, for instance, when the AI makes the wrong decision or is suddenly not able to handle the driving task. As a practical example, let us think of a situation when advanced Lane Assistance is enabled but, suddenly, the car is not able to read the road lane signage anymore. Therefore, it becomes paramount to monitor not only the surroundings but also the drivers' psychophysical state in order to be sure that it would be able to promptly intervene. In other words, AI has to be based on a user-centered approach.

To this regard, the present study aimed to investigate a specific use case, i.e., driving mental fatigue that can dramatically impact the drivers' capacities to intervene and take over the car control, especially after periods of high automation that promote the out-of-the-loop phenomenon (Endsley and Kiris, 1995; Di Flumeri et al., 2019).

To achieve this objective, participants were engaged in a 45-min-long monotonous (i.e., across a repetitive urban path at a slow speed without traffic) driving task after a 15-min-long high-demanding driving task. The preliminary driving task was aimed at “stressing” the drivers in order to promote the onset of fatigue in the following task, according to what was suggested by scientific literature (Thiffault and Bergeron, 2003; García et al., 2010). The analysis of the questionnaires validated such experimental design since the participants claimed to feel significantly more fatigued at the end, resulting in a higher score for both KSS and Chalder questionnaires (Figure 2). Asking participants to rate both perceived fatigue and sleepiness might seem redundant. However, it has to be considered that fatigue and sleepiness are often hard to self-assess. Also, they are contiguous phenomena. While sleepiness is mainly defined as the tendency to fall asleep and is quite easy to subjectively recognize, fatigue is a more undefined concept (Shen et al., 2006; Shahid et al., 2010). In particular, considering that the aim of the paper was to intercept the early stages (i.e., onset) of fatigue and for this reason a not excessively exhausting protocol was adopted and considering also the high inter-individual variability in terms of fatigue resistance, the perception of the mental state induced in the participants was not obvious.

From the analysis of neurophysiological parameters during the monotonous driving task, three parameters, in particular, have been demonstrated to be sensitive to increasing fatigue, namely, the EEG-based MDrow index, the Eye Blink Rate, and the Eye Blink duration. In particular, the former significantly increased over time, as expected from previous validations (Ronca et al., 2022), with higher values in the second (16–30 min) and the third (31–45 min) segments with respect to the first one (1–15 min) (Figure 4). Ocular blink-related parameters showed a significant effect as well, with an increased Eye Blink Rate only in the third segment (Figure 5A) and decreased Eye Blink Duration (Figure 5B) in all the segments. Despite some previous findings in the scientific literature (Bundele and Banerjee, 2009; Fujiwara et al., 2018; Alaimo et al., 2020), no significant effects were found on the other investigated physiological parameters.

This group analysis points out a first relevant outcome: the physiological parameters do not react with similar time dynamics, for instance, the EEG-based parameters can highlight the onset of fatigue in advance with respect to ocular parameters. This is crucial information that is not possible to obtain if all the data are not recorded simultaneously. This is also relevant information to consider when developing systems that employ a multimodal approach to increase their sensitivity to the phenomenon: if the information is not aligned over time, proper countermeasures have to be investigated and developed; otherwise, the combination of multimodal data will become a weakness more than a strength for the system.

The analysis of behavioral data, in terms of Reaction Times, pointed out another relevant concern: it seems that mental fatigue appeared with a different timing across participants. In fact, it can be assumed that during a repetitive task, the human Reaction Times to an external event should improve over time because of habituation (Mackworth, 1968), but it cannot happen if interference in terms of mental impairment, such as mental fatigue, appears (Lorist et al., 2005). On the basis of this assumption, whether the sample showed different behaviors was investigated; actually, while one group did not improve its RT performance over time, the second one did; therefore, these findings can be interpreted as the fact that Group 1 experienced mental fatigue earlier, while the Group 2 remained alert for almost all the task and maybe was mentally fatigued only at the end. The presence of a significant interaction effect supported this assumption (Figure 6). We are conscious that this is a speculative hypothesis but it could explain the difference we found in behavioral response (RT) as well as the difference found in some neurophysiological parameters. Indeed, after observing these different behavioral responses, the neurophysiological indicators were analyzed again by including RT as a grouping variable; the results observed did not refute this interpretation. While Group 1, the early fatigued group, showed a significant increase in the EEG-based index in the second segment, Group 2 showed a similar increase only in the third segment, i.e., at the end (Figure 7). At the same time, in terms of ocular parameters, Group 1 showed an EBR significant increase during the third segment, while Group 2 showed an increasing trend but without statistical significance. In other words, these results confirmed what was found in the overall sample: the ocular parameters look to be sensitive to mental fatigue, but the effect is delayed with respect to the EEG parameters.

Also, this outcome points out another important concern, i.e., interindividual variability in terms of fatigue experience. Some of the previous studies in literature, as well as some preliminary attempts made by automotive industries, were based on data recording from large populations: the participants are usually asked to drive for 2 h, and the last 30 min were assumed a-priori as representative of a “fatigued” behavior. The results of the present study underline that such a-priori hypothesis could not be correct, potentially leading to inaccurate results. The neurophysiological monitoring of the driver could allow for a more precise and comprehensive overview of the driver's actual behavior and the mental causes, not only with respect to fatigue but also to other impairing mental conditions, such as decreasing vigilance and inattention.

The main limitation of the study is the average long time windows, i.e., the 15-min-long segments that could have reduced eventual effects related to other physiological parameters such as heart activity and skin sweating, previously found in the literature. However, the choice of adopting such a window was made in order to keep the statistic robust regardless of the sample size; the whole task was divided into three equal segments because with 26 observations (the participants), more segments would have reduced the statistical power. A further limitation is represented by the limited duration of the driving task (one hour of driving in total). Future implementations of the study should take into consideration a longer driving protocol in order to induce and observe fatigue in those participants who are more fatigue-resilient. A temporal dynamic of fatigue occurrence should be studied, also considering the interplay of subjective response with the intervention of AI driving the car. Indeed, different levels of automation (high or low) could affect the onset of fatigue, anticipating or delaying it. In the current paper, we decided to fix the gender variable by recruiting only men in order to not introduce a confounding variable during the analysis. Our choice is also motivated by the actual gender balance among professional drivers. But even if nowadays professional drivers are mostly men [94–98% among commercial good drivers (New IRU Survey Shows Driver Shortages to Soar., 2021; Scott and Davis-Sramek, 2023)], future studies involving professional drivers should also recruit female participants to investigate any possible gender difference in the temporal dynamic of fatigue insurgence as well as in its neurophysiological correlates.

From a technological point of view, it will be crucial for the scientific community, as well as the industry, to investigate how to deal with the different time responsiveness of the different parameters. In fact, AI methodologies usually benefit from a large amount of data. A multimodal approach constituting relevant data sources, i.e., in our case, the time series of the different neurophysiological parameters, will undoubtedly improve the effectiveness and the reliability of a model aimed at making decisions on the basis of its situation assessment. On the other hand, it is crucial that the information is “synchronized” across the different data sources instead of using a single data source; otherwise, their fusion could have a negative more than a positive effect. Further research on this topic is encouraged. It should be taken into consideration that in a real application of this monitoring method, Reaction Times could not be used as they were in this paper. An AI predictive model in real environments needs clear and defined measures in order to prevent dangerous situations. Asking drivers to perform a task in order to collect behavioral data (i.e., RT) is not safe. Also, drivers could omit a response (because of inattention or because of a safety matter). AI relying on this kind of response would not be reliable. Another adoption of RT that is compatible with the application of this monitoring system in a real scenario could be during the calibration of the AI system. RT could be used to evaluate the subjective neurophysiological parameters related to a fatigued mental state in order to set a threshold for each individual driver. In this way, the system could perform profiling of the drivers to produce individual feedback regarding their state allowing more accurate monitoring.

Also, good quality of data collection must be ensured. In this paper, we had 21% of data loss (EEG and EOG) which is an acceptable level in ecological conditions. Future studies should investigate the source of data loss which could probably be addressed by analyzing the driving style of the drivers, leading to movement artifacts. To do so, driving parameters coming from the vehicle could help in figuring out how to minimize data corruption while collecting the data.

Lastly, but not less important, it could be argued that despite the promising results, a user-centered AI is still far from being deployed in real contexts because of the invasiveness of the neuromonitoring systems. However, we believe that the recent progress in terms of wearable technology (Ronca et al., 2023) will help to overcome this limitation very soon.

## 5 Conclusions

The present study aimed to investigate, in the specific use case of driving mental fatigue, whether neurophysiological parameters can be used to assess drivers' mental fatigue online, in order to enable a new “sensing” channel toward the drivers. The expected impact is to make the vehicle AI aware not only of the surroundings but also of the drivers' psychophysical state in order to continuously check their ability to take over car control during the current scenario where the driving responsibility is and will be still shared between the human driver and AI.

By means of a holistic approach, considering simultaneously a large set of neurophysiological parameters, in particular electroencephalographic, electrooculographic, photopletismography, and electrodermal activity data, it was possible to determine which neurophysiological parameters could help to achieve the overarching aim of the study.

Results showed that the most sensitive and timely parameters are those related to brain activity. To a lesser extent, those related to ocular parameters are also sensitive to the onset of mental fatigue but with a delayed effect.

In conclusion, it would be possible to effectively monitor drivers' mental fatigue in real-time in order to feed the AI vehicle with driver-related information; the challenge now is how to deal with the different time dynamics of the phenomena. Further research on the implementation of this concept is encouraged.

## Data availability statement

The raw data supporting the conclusions of this article will be made available by the authors, without undue reservation.

## Ethics statement

The studies involving humans were approved by the Sapienza University of Rome RomaTre University of Rome. The studies were conducted in accordance with the local legislation and institutional requirements. The participants provided their written informed consent to participate in this study. Written informed consent was obtained from the individual(s) for the publication of any potentially identifiable images or data included in this article.

## Author contributions

Conceptualization: AG, GD, VR, and CP. Methodology: GD, PA, and GB. Formal analysis: AG, VR, AV, RC, LT, and IS. Investigation: GD, AG, and MA. Resources: SS, MP, RV, and MG. Data curation: AG, AB, and RC. Writing—original draft preparation: AG and VR. Writing—review and editing: AV, IS, LT, and AB. Funding acquisition: MG, FB, CP, and MP. All authors have read and agreed to the published version of the manuscript.
